# Slow‐Release Formulation of Cowpea Mosaic Virus for In Situ Vaccine Delivery to Treat Ovarian Cancer

**DOI:** 10.1002/advs.201700991

**Published:** 2018-02-21

**Authors:** Anna E. Czapar, Brylee David B. Tiu, Frank A. Veliz, Jonathan K. Pokorski, Nicole F. Steinmetz

**Affiliations:** ^1^ Departments of Pathology Case Western Reserve University 2109 Adelbert Road Cleveland OH 44106 USA; ^2^ Department of Biomedical Engineering Case Western Reserve University 2109 Adelbert Road Cleveland OH 44106 USA; ^3^ Departments of Macromolecular Science and Engineering Division of General Medical Sciences‐Oncology Case Western Reserve University 2109 Adelbert Road Cleveland OH 44106 USA; ^4^ Departments of Materials Science and Engineering Division of General Medical Sciences‐Oncology Case Western Reserve University 2109 Adelbert Road Cleveland OH 44106 USA; ^5^ Departments of Radiology Division of General Medical Sciences‐Oncology Case Western Reserve University 2109 Adelbert Road Cleveland OH 44106 USA; ^6^ Departments of Case Comprehensive Cancer Center Division of General Medical Sciences‐Oncology Case Western Reserve University 2109 Adelbert Road Cleveland OH 44106 USA

**Keywords:** cancer immunotherapy, cowpea mosaic virus, in situ vaccines, slow release therapeutics, viral nanoparticles

## Abstract

The plant viral nanoparticle cowpea mosaic virus (CPMV) is shown to be an effective immunotherapy for ovarian cancer when administered as in situ vaccine weekly, directly into the intraperitoneal (IP) space in mice with disseminated tumors. While the antitumor efficacy is promising, the required frequency of administration may pose challenges for clinical implementation. To overcome this, a slow release formulation is developed. CPMV and polyamidoamine generation 4 dendrimer form aggregates (CPMV‐G4) based on electrostatic interactions and as a function of salt concentration, allowing for tailoring of aggregate size and release of CPMV. The antitumor efficacy of a single administration of CPMV‐G4 is compared to weekly administration of soluble CPMV in a mouse model of peritoneal ovarian cancer and found to be as effective at reducing disease burden as more frequent administrations of soluble CPMV; a single injection of soluble CPMV, does not significantly slow cancer development. The ability of CPMV‐G4 to control tumor growth following a single injection is likely due to the continued presence of CPMV in the IP space leading to prolonged immune stimulation. This enhanced retention of CPMV and its antitumor efficacy demonstrates the potential for viral–dendrimer hybrids to be used for delayed release applications.

## 1 Introduction

Ovarian cancer is the leading cause of death among gynecologic malignancies in the United States. While platinum chemotherapies are usually initially effective in treating ovarian cancer, the development of platinum resistance often leads to disease recurrence, making clear that novel and more effective treatment strategies are needed.^1^ Immunotherapies hold great promise and a variety are currently under investigation for treatment of ovarian cancer and other malignancies.^2^, ^3^, ^4^, ^5^ We recently demonstrated the efficacy of an in situ vaccination strategy using the nanoparticles formed by the plant virus cowpea mosaic virus (CPMV); efficacy was demonstrated in several tumor types, including ovarian cancer.^6^ An in situ vaccine works through direct administration of an immune‐stimulatory agent, here CPMV, into the local tumor environment to reverse tumor‐mediated immunosuppression and resensitize the immune system to tumor specific antigens. A particular advantage of this approach is that in situ vaccines are not limited by the presence of known antigens in tumor tissue.^7^ The in situ vaccine triggers innate and adaptive antitumor responses, where the tumor itself serves as the antigen source eliminating the need to determine specific antigens for a given malignancy.^7^, ^8^

In our previous study we demonstrated that in a syngeneic, orthotopic mouse model of ovarian cancer, weekly intraperitoneal (IP) treatment using the CPMV in situ vaccine significantly delayed tumor growth and improved survival.^6^ Injection of CPMV into cancer tissue induced a potent immune response that is thought to be related, in part, by conversion of the resident immune suppressive neutrophils present in tumors to pro‐inflammatory type neutrophils. These activated neutrophils are capable of both directly killing tumor cells and priming T cells and NK cells toward antitumor activity.^6^ While IP infusions of chemotherapies are currently approved for use clinically, and have been shown to provide a survival advantage in the treatment of ovarian cancer, this method of administration is underutilized. In contrast to intravenous (IV) administration that can be done in the out‐patient setting, IP administration often requires a hospital admission leading to increased cost and decreased quality of life.^9^, ^10^ Therefore, to alleviate the need for repeat administration, we set out to develop a slow‐release formulation of the CPMV in situ vaccine that would maintain sustained immune stimulation without the need for repeat IP injections. Maintaining an immunostimulatory effect through depot formation could reduce the number of IP administrations, making this treatment strategy more attainable for clinical implementation.

A number of slow‐release formulations of cancer drugs and immunotherapies are currently under investigation;^11^, ^12^ these include hydrogels for delayed release of tumor associated antigens,^13^ chemotherapeutics,^14^ and a variety of other cargo including nucleic acids,^15^ as well as microparticle^11^ and microneedle^16^ administration of antibodies for immunotherapy. These diverse approaches all seek to improve cancer treatment efficacy by maintaining a constant therapeutic concentration and improve cancer patient quality of life by reducing the number of administrations.

In this work, we aimed to develop slow‐release assemblies of CPMV making use of charged dendrimers and electrostatic self‐assembly protocols. While zwitterionic in nature, CPMV carries an overall negative surface charge, therefore we chose to program coassembly with positively‐charged polyamidoamine (PAMAM) dendrimers. A variety of different dendrimer formulations are under investigation for use in drug delivery and nanomedicines; they have the advantage of being highly tunable, multivalent, and possessing a low polydispersity.^17^ In addition to their use in drug delivery, dendrimers have been used in combination with oncolytic adenovirus in order to improve intratumoral accumulation^18^ and in virus–dendrimer assemblies as a method to create well‐controlled higher order structure on a nanoscale‐to‐mesoscale level.^15^ PAMAM dendrimers in particular are well‐understood, uniform, and commercially available; our lab has successively used them in the past to create virus–dendrimer hybrid materials for photon capture applications.^19^ The potential for in situ vaccination applications using virus–dendrimer hybrids however, has not yet been fully explored. Therefore, we investigated the assembly and disassembly of CPMV–dendrimer hybrids, their IP trafficking, and efficacy of treatment using them in a mouse model of ovarian cancer.

## 2 Results and Discussion

### 2.1 Formation of the CPMV‐G4 Assembly

CPMV was propagated in and isolated from black‐eyed pea plants.^20^ Molecular farming of pharmaceuticals in plants is advantageous because plant‐based production avoids contamination with mammalian pathogens or contaminants such as endotoxins. In recent years, plant virus‐based nanotechnologies have been recognized for their possible applications in human health. Their well‐defined nanoscale structures in combination with genetic and chemical programmability make them an attractive platform technology.^21^, ^22^ CPMV is the type member of the comovirus genus in the family *Comoviridae*. Members of this family are also known as plant picorna‐like viruses as they share similarities in structure, genome organization, and replication strategy with animal picornaviruses. CPMV infects legumes and was first reported in *Vigna unguiculata*. In systemic infected plants CPMV typically causes mosaic or mottling symptoms.^23^ CPMV has a bipartitite positive‐sense RNA genome encapsidated separately in isometric, icosahedral nanoparticles measuring 30 nm in diameter. The structure of CPMV is known to near atomic resolution. The virions are formed by 60 copies each of a small and large coat protein yielding a pseudo *T* = 3 symmetry.^24^

**Figure 1:**
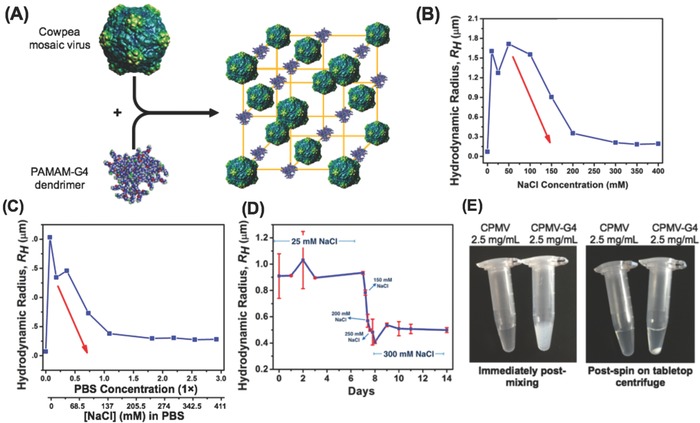
A) Schematic illustration of the assembly of CPMV with PAMAM‐G4 dendrimer (space model with minimized energy calculated using Spartan software). DLS measurements for the assembly and disassembly of CPMV and PAMAM‐G4 dendrimer in B) increasing NaCl and C) PBS concentrations. D) Stability study for the CPMV‐G4 assembly, wherein the ionic strength was initially fixed at 25 × 10^−3^ m NaCl for 1 week, gradually increased, and was kept at 300 × 10^−3^ m NaCl for another 7 d. E) Aggregates of high concentration (2.5 mg mL^−1^) CPMV and PAMAM‐G4 in 25 × 10^−3^ m NaCl, UV–vis spectroscopy of the supernatant from these aggregates was found to be below the linear range for detection of protein in solution indicating that there is minimal CPMV remaining in the solution (Figure S1, Supporting Information).

While the CPMV structure, like any proteinaceous macromolecule, is zwitterionic and patchy in nature, the electrostatic surface map and zeta potential measurement indicate that the net surface charge is negative (ζ = −12 mV), thus enabling coassembly with positively charged polymers.^19^ **Figure** **1**A depicts the overall assembly scheme; the electrostatically driven assembly of the CPMV nanoparticles with the amine‐terminated generation 4 PAMAM dendrimer was investigated through dynamic light scattering (DLS) measurements. Combining both, CPMV and G4, at a concentration of 0.15 mg mL^−1^ each in pure MilliQ water immediately resulted to an increase in hydrodynamic radius *R*_H_; from 15 nm (for CPMV), 56% of the assembly increased to an *R*_H_ of 71 ± 5 nm, while 44% measured to be 841 ± 363 nm. Increasing the ionic strength to 10 × 10^−3^ m NaCl abruptly induced the formation of larger aggregates with diameters greater than 1.5 µm (97% of the assemblies). These large aggregates, which constituted at least 96% of the assemblies, were observed at salt concentrations up to 150 × 10^−3^ m; higher concentrations gradually disassembled the CPMV‐G4 aggregates, with concentrations greater than 200 × 10^−3^ m forming aggregates with two *R*_H_ distributions averaging at 10–13 nm (25% of the assemblies) and 200–350 nm (75–78% of the assemblies) (Figure 1B). A similar trend was observed when a related experiment was conducted with increasing phosphate buffered saline (PBS) concentration. From 0.07 to 0.36 × PBS, which corresponds to approximately NaCl concentrations of 10 to 50 × 10^−3^ m, 95–99% of the assemblies reached an *R*_H_ between 1.3 to 2 micrometers; less than 5% of the assemblies had diameters less than 150 nm. The hydrodynamic radius of the assemblies gradually decreased starting at PBS concentrations higher than 0.36 ×, while other smaller aggregates with *R*_H_ < 10 nm start to be observed as well (Figure 1C). At 3.65 × PBS, 7% of the assemblies have an *R*_H_ of 14 nm, which may correspond to CPMV; 65% resulted to 2.46 nm which could be aggregated PAMAM dendrimers; only 30% of the assemblies maintained an *R*_H_ of ≈250 nm. Complete list of the average hydrodynamic radius values, polydispersity index and percent composition of the aggregates in NaCl and PBS environments are presented in Tables S1 and S2 in the Supporting Information. These data indicate that the presence of a low salt concentration promotes formation of larger aggregates; while CPMV and the G4 dendrimers still have some interaction at zero salt concentration, as indicated by the measured hydrodynamic radius of 70 nm for 56% of the assembly, the repulsion between the positively charged G4 limits greater aggregation in the absence of ions for some electrostatic shielding. When the salt level is increased too high however, salt screening effects limit the interactions between the dendrimers and CPMV, thus reducing overall aggregate size. These properties are thought to be useful for in vivo biomedical applications, i.e., the use of slow‐release formulation: at low salt the assembly is triggered, and postinjection into the tissue, under physiologic salt concentrations (136–145 × 10^−3^ m),^25^ disassembly and CPMV release is induced.

The stability of the CPMV‐G4 assembly was investigated by storing the mixture in a low ionic strength environment (25 × 10^−3^ m NaCl) for a week, disassembled by gradually increasing the salt concentration, and held in high ionic strength conditions (300 × 10^−3^ m NaCl) for another week. The assembly maintained its large hydrodynamic radius at low salt concentrations for 7 d. Exposure to higher ionic concentrations (150–300 × 10^−3^ m NaCl) led to a burst release into smaller aggregates with an *R*_H_ of 500 nm; then slow decrease in size that may indicate slow‐release of CPMV from the smaller aggregates (Figure 1D).

For animal studies, the concentration of CPMV was increased to 2.5 mg mL^−1^ to form larger aggregates and reduce the total volume of injection necessary, this concentration resulted in aggregates large enough to immediately become visible upon mixing but still capable of being resuspended in solution and passed easily through a syringe. The CPMV:G4 ratio was maintained at 1:1 and salt concentration was kept low (25 × 10^−3^ m NaCl). Due to the high concentration of CPMV and G4, large aggregates formed immediately and *R*_H_ could not be determined by DLS, supporting that the aggregates were at least larger than 2.5 µm. A cloudy appearance was observed immediately and aggregates were collected at the bottom of the tube following a brief spin using a tabletop centrifuge following a 30 s spin in a tabletop centrifuge at 15 000 × g, indicating successful formation of CPMV–dendrimer aggregates (Figure 1E). UV–vis spectroscopy of the supernatant from these aggregates was found to be below the linear range for detection of protein in solution indicating that there is minimal CPMV remaining in the supernatant and the overwhelming majority of CPMV interacts with PAMAM‐G4 to form the visible aggregates (Figure S1, Supporting Information). The colloidal suspension was mixed well prior to use in animals.

**Figure 2:**
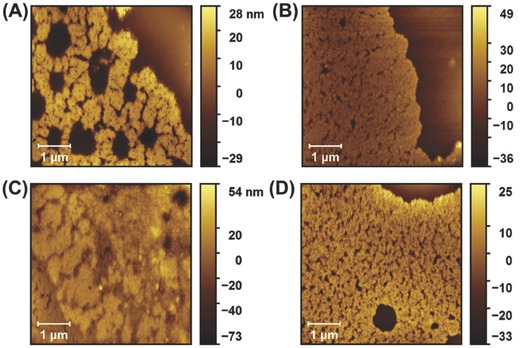
Topographical tapping‐mode AFM images of A) 0.15 mg mL^−1^ CPMV and 0.15 mg mL^−1^ CPMV‐G4 (1:1) in B) 0 × 10^−3^ m, C) 50 × 10^−3^ m, and D) 150 × 10^−3^ m NaCl, which were dropcasted on a freshly cleaved mica surface.

The morphologies of the assemblies were further investigated through tapping mode‐atomic force microscopy (AFM). A 0.15 mg mL^−1^ solution of CPMV, in the absence of the G4 dendrimer, shows areas of packed CPMV when cast on a freshly cleaved mica surface, however the presence of large empty spaces demonstrates that there are not strong interactions between the nanoparticles (**Figure** **2**A). In contrast, when 0.15 mg mL^−1^ CPMV is mixed with G4 at a 1:1 ratio in a salt‐free solution, particles form large areas of tightly packed CPMV nanoparticles on the surface (Figure 2B). Increasing the ionic concentration to 50 × 10^−3^ m NaCl induced formation of stacks of packed CPMV nanoparticles (Figure 2C), indicating further increased interactions between CPMV and the G4 dendrimers. At higher ionic concentration (150 × 10^−3^ m NaCl) a layer of tightly arranged CPMV formed, similar to the formation observed in the absence of salt (Figure 2B,D), supporting the conclusion that high salt concentrations reduce formation of large aggregations. Overall, the morphological variations of the CPMV‐G4 assembly correlate to the DLS hydrodynamic radius measurements, which exhibit the responsive assembly and disassembly of the electrostatic interactions as a function of salt concentration.

These findings are in agreement with previous work showing that increasing salt concentrations reduce the size of aggregate formation through reducing electrostatic interactions between dendrimers and viral nanoparticles.^26^, ^27^, ^28^ While salt concentration has been known to be important in the assembly formation of virus–dendrimer hybrid materials, to our knowledge, the disassembly of these hybrid materials has not been investigated or utilized for biomedicine.

Together, our data indicate that while the larger aggregates are stable for extended periods of time at low salt concentrations, the larger structures disassemble at ionic concentrations found in the IP space, sodium concentrations in the IP space are similar or slightly lower than found in serum (136–145 × 10^−3^ m).^25^, ^29^ Even at the highest salt concentration measured however, CPMV‐G4 aggregates would still be too large to passively diffuse across blood vessels to enter systemic circulation from the intraperitoneal space (size limit < 100 nm) and would require disassembly or lymphatic drainage for eventual clearance.^30^ Further, the slow time course of disassembly of the aggregates at physiological salt concentrations (Figure 1C), makes this assembly a promising candidate for continued presence in the IP space with slow release over time.

### 2.2 Treatment of Ovarian Cancer in a Syngeneic Mouse Model

**Figure 3:**
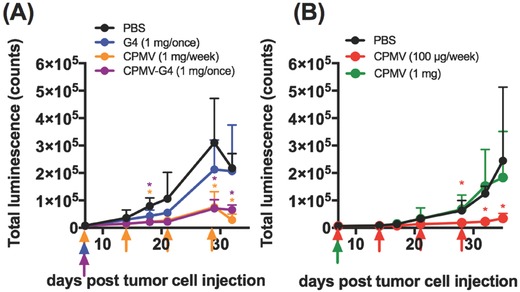
Growth of ovarian cancer cells in the intraperitoneal space. Treatment was injected intraperitoneally 7 d following injection of 2 million luciferase‐positive ID8‐*Def29/Vegf‐A* cells. Injection of each treatment group is indicated with a color‐coordinated arrow. PBS was also injected weekly. Total bioluminescence was measured in intraperitoneal space until ascites development was apparent in the PBS control group (*n* = 5–6). PBS/weekly in black, G4/1 mg once (normalized to the amount of G4 in CPMV‐G4) in blue, CPMV‐G4/1 mg once in purple, CPMV/1 mg weekly in orange, CPMV/100 µg weekly in red, CPMV/1 mg once in green. Graphs represent two successive experiments. (*p < 0.05 as calculated by Excel two‐tailed t‐test when treatment group is compared to PBS, no statistical difference was found was between G4 only and PBS or a PBS and a single injection of CPMV 1 mg).

Treatment efficacy of the CPMV‐G4 formulation was assessed using an aggressive, syngeneic, and orthotopic mouse model of ovarian cancer.^31^ To monitor tumor development, we transfected the cells with luciferase.^32^ Treatment was started 1 week following injection of 2 million ID8‐*Defb29/Vegf‐A‐Luc* cells into the IP space using female C57BL/6 mice. Establishment of disease was confirmed and then monitored using in vivo imaging system (IVIS) imaging. Groups were treated with the following treatments: a single dose of 1 mg of CPMV versus a single dose 1 mg CPMV equivalent of CPMV‐G4 versus 100 µg of free CPMV administered weekly (100 µg per week is the dosing previously shown to be effective at treating ovarian cancer growth^6^); intraperitoneal PBS was administered as a control. An additional study compared 1 mg PAMAM‐G4 alone to 1 mg CPMV equivalent of CPMV‐G4. A dose of 1 mg was chosen for delayed release formulations as it provides a high enough initial dose for continuous release while still being well tolerated in treated animals. Disease burden was monitored twice weekly by tracking total luminescence indicating cancer cell growth. As it is difficult to directly compare tumor growth curves in studies not conducted at the same time these studies are plotted separately (**Figure** **3**A,B).

Tumor burden in mice treated with a single dose of CPMV‐G4 (1 mg) was very similar to total luminescence observed in mice treated weekly with soluble CPMV (100 µg), and both treatment groups had significantly delayed cancer cell growth (Figure 3). In contrast, a single 1 mg dose of soluble CPMV did not slow tumor growth, indicating that not the total dose but the treatment schedule, (maintaining the therapeutic CPMV concentration within the IP space over prolonged periods), is critical (Figure 3). The G4 administered alone did not have a significant effect on tumor growth—while not statistically significant, there is trend that tumors grow slower in mice treated with G4, likely due to nonspecific cytotoxicity and immune stimulation associated with PAMAM dendrimers.^33^, ^34^

Together these studies indicate that CPMV‐G4 assemblies can function as depots for slow‐release of the CPMV in situ vaccine, prolonging its therapeutic efficacy in a mouse model of ovarian cancer after a single IP administration. The retention of CPMV when administered as soluble versus assembled formulation in the IP space was further tested by examining the biodistribution of fluorescently labeled CPMV and CPMV‐G4.

### 2.3 Biodistribution of CPMV‐G4 Administered Intraperitoneally

**Figure 4:**
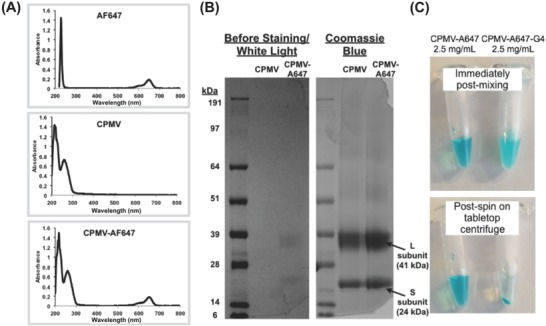
Characterization of dye‐labeled CPMV and CPMV‐G4 assemblies. A) Representative UV–vis absorption spectra of AF647, CPMV, and CPMV‐AF647 particles. B) SDS‐PAGE analysis of wild type CPMV and CPMV‐AF647 in white light and after Coomassie Blue staining. C) Free CPMV‐AF647 and aggregates of CPMV‐AF647‐G4 immediately following mixing and after a brief spin in a table‐top centrifuge (aggregates are too large to be measured with DLS).

The biodistribution and clearance rate of CPMV compared to CPMV‐G4 was evaluated using in vivo fluorescence imaging (Spectrum BLI). Mice without tumors were injected with 1 mg of CPMV labeled with AlexaFluor 647 either as free CPMV or formulated as the CPMV‐G4 assembly as described above. CPMV nanoparticles were covalently functionalized with NHS‐AF647 dye molecules using the exposed lysine residues on the capsid surface. The successful chemical labeling of the CPMV particles post purification was verified using UV–vis absorption spectroscopy; an average of 40 dye molecules per CPMV were attached (**Figure** **4**A). A sodium dodecyl sulfate (SDS) gel electrophoresis was also conducted to confirm the covalent conjugation of AF647 dye molecules to the CPMV coat proteins (Figure 4B). Under white light, bright blue bands were observed matching the small and large subunit proteins of CPMV‐AF647, which was not evident for CPMV; staining with Coomassie Blue, shows that CPMV coat proteins are colocalized with the dye. Successful assembly of fluorescent AF647‐CPMV‐G4 formulation was confirmed by immediate visual changes in the mixture as well as accumulation in the bottom of a centrifuge tube following a 30 s spin on a tabletop centrifuge (at 15 000 g) (Figure 4C).

**Figure 5:**
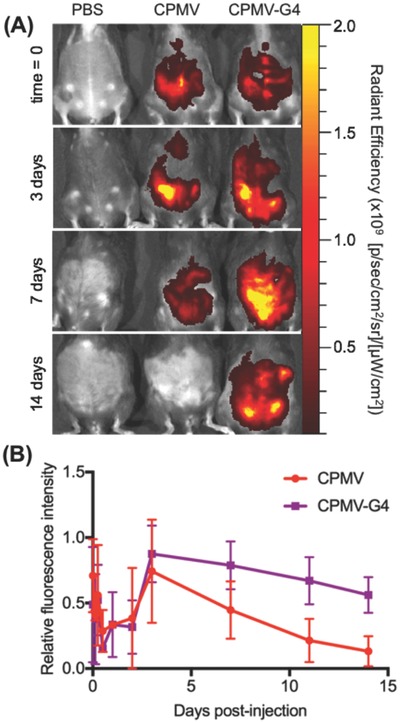
Retention of CPMV and CPMV‐G4 in the intraperitoneal space. A) Fluorescence imaging of C57/BL6 mice immediately following injection, on days 3, 7, and 14. B) Normalized fluorescent intensity as determined by ROI analysis; the highest measured fluorescence intensity in the intraperitoneal space over the course of the study was established as 1 and relative intensity for all subsequent time‐points was calculated as a portion of this intensity (*n* = 3).

Biodistribution was then determined using a longitudinal imaging approach; at defined time points over a 14 d course, total fluorescence in the IP space was determined using Spectrum BLI and region of interest (ROI) analysis (**Figure** **5**). Free CPMV was cleared from the IP space more quickly than CPMV‐G4; ≈50% of the maximal measured fluorescence intensity was present for free CPMV at 7 d, in contrast over 50% of the maximal measured fluorescence intensity in CPMV‐G4 treated animals persisted at day 14. Overall CPMV‐G4 has roughly a 3 fold longer half‐life in the peritoneum than free CPMV. Both groups showed an initial increase in measured fluorescent intensity, likely due to a reduction in the fluorescence quenching of the fluorophores;^35^ perhaps as the CPMV spread throughout the IP space, the overall concentration of fluorophores was reduced and measured fluorescence increased. To prevent this early quenching from causing artificially high percent retention to be calculated, relative fluorescence intensity was calculated from the maximum fluorescence measured and not the initial intensity measurement. While this quenching effect limits the utility of in vivo fluorescent imaging to determine the absolute concentration of CPMV still present in the IP space, it still provides useful information as to the relative speed at which free CPMV and CPMV‐G4 assembly is cleared and supports enhanced retention of CPMV in the CPMV‐G4 assembly compared to free CPMV.

These biodistribution studies are in good agreement with the efficacy studies that show a single administration of CPMV‐G4 was as effective as weekly injections of free CPMV (Figure 3). This confluence of data supports the conclusion that CPMV, when formulated as CPMV‐G4 assemblies, forms a depot and is detachable over prolonged periods of time, continually stimulating antitumor immunity. For this depot strategy of in situ vaccination to be effective, the depots must be retained within the peritoneal site of ovarian cancer. We hypothesize that the larger CPMV‐G4 aggregates are not easily cleared through leaking into blood vessels or lymphatic drainage in the IP space without disassembly.

Compared to other systems, the observed retention time for CPMV‐G4 is longer; for example polymer‐based hydrogels injected intraperitoneally were found to persist at 8 d following injection.^36^ Further, while hyaluronic acid based hydrogels have been shown to persist in the IP space for a comparable or longer time, their use as a drug carrier has led to both decreased^37^, ^38^ and increased tumor growth,^39^ indicating that further investigation of this system, especially its degradation byproducts, is needed before it can be considered for use in slow‐release formulations. Finally, our system has several advantages over other types of slow‐release formulations including implantable devices that typically require an invasive surgery;^40^ the virus–dendrimer colloidal solution is nonviscous and can be administered with a syringe without surgical intervention. As with any nanotechnology, the advantages and disadvantages must be carefully considered; unlike plant‐virus based materials, PAMAM dendrimers have been shown to be toxic at high concentrations and further study of the in vivo properties of dendrimers and associated hybrid materials is still needed in order to better understand associated toxicity and interaction with the immune system.^33^, ^34^, ^41^ Future studies may explore the assembly with other polymeric systems.

## 3 Conclusions

Virus–dendrimer hybrid materials are a novel class of materials with a number of potential applications targeting materials and human health. Here we present the application of the CPMV‐G4 hybrid assembly as an immunotherapy; specifically the assembly functions as a depot with slow release of the immunostimulatory nanoparticle CPMV in the IP cavity, therefore prolonging its therapeutic antitumor effect in a mouse model of IP disseminated ovarian cancer. A single administration of the CPMV‐G4 hybrid resulted in matched efficacy compared to weekly treatment using the soluble CPMV formulation. This is an important finding and may enable the in situ vaccine application of CPMV assemblies for difficult to inject tumors, such as ovarian cancer or gliomas; in these disease settings reducing the number of necessary administrations while maintaining a potent immunotherapy effect is an important goal to enable successful translation of in situ applied (immune)therapeutics.

## 4 Experimental Section

*Preparation of CPMV Nanoparticles and Generation 4 PAMAM Dendrimer*: CPMV was propagated in black‐eyed pea plants (*V. unguiculata)* and isolated using previously reported protocols.^42^

For biodistribution studies, the CPMV particles were conjugated with an Alexa Fluor 647 dye (NHS‐AF647) using a previously described reaction.^35^ In brief, dye labeling was carried out overnight at a concentration of 2 mg mL^−1^ CPMV in 0.1 m potassium phosphate buffer (pH 7.0) and 10% DMSO with an excess of 2000 dyes per CPMV. The dye‐functionalized CPMV particles (CPMV‐AF647) were purified by ultracentrifugation and characterized using UV–vis absorption spectroscopy using a Nanodrop instrument. Dyes per particle were determined using the extinction coefficients and absorption maximums 8.1 mg^−1^cm^−1^ at 260 nm for CPMV^35^ and 270 000 m ^−1^ cm^−1^ at 650 nm for AF647 (per company website). Attachment was confirmed with electrophoresis using 4–12% NuPage bis‐tris gels (Invitrogen).

Generation 4 PAMAM dendrimers with an ethylenediamine core (10 wt% in methanol) used in the study was purchased from Sigma Aldrich. PAMAM‐G4 was isolated by removing methanol through rotary evaporation and resuspended in MilliQ water at a 10 mg mL^−1^ concentration.

*Dynamic Light Scattering*: To study the assembly and disassembly of CPMV‐G4 DLS measurements were performed: CPMV (≈50 mg mL^−1^ in 0.1 m PBS, pH 7.0) and PAMAM‐G4 dendrimers (10 mg mL^−1^ in MilliQ water) were mixed at 0.15 mg mL^−1^ of virus and 0.15 mg mL^−1^ dendrimer concentration. The ionic strength was modified by adding small amounts of either 2 m NaCl or 10 × PBS stock solutions. The hydrodynamic radius of the virus–polymer assemblies was measured using a DynaPro Nanostar DLS instrument (Wyatt Technology, Goleta, CA) at a wavelength of 658 nm, 90° scattering angle, 25 °C.

*Atomic Force Microscopy*: The morphology of the CPMV‐G4 assemblies was imaged using a 5500 atomic force microscope (Keysight Technologies, Inc., formerly Agilent Technologies) in tapping mode. High‐resolution noncontact gold‐coated NSG30 silicon cantilevers (NT‐MDT Spectrum Instruments, Tempe, AZ) with a resonant frequency of 240–440 kHz were used and mounted onto the piezoelectric scanner for AFM imaging. The resulting AFM images were processed using Gwyddion Ver. 2.47.

*Ovarian Cancer In Vivo Efficacy and Biodistribution*: Animal studies were carried out using IACUC‐approved protocols. Female C57BL/6 mice (Jackson Labs) were injected intraperitoneally with 2 million cells of the highly aggressive, luciferase‐positive, murine ovarian cancer cell line ID8‐*Defb29/Vegf‐A* in sterile PBS. ID8‐*Defb29/Vegf‐A* cells were transfected with luciferase as previously described.^32^ Cancer cell growth was monitored using the Perkin Elmer IVIS Spectrum; mice were injected intraperitoneally with luciferin (15 mg mL^−1^, 150 µL intraperitoneally) and imaged 5 min postinjection with a 3 min exposure time. Total luminescence was determined using Living Image software and total counts per mouse were graphed. Treatment was initiated 7 d following cell injection and administered either weekly (PBS and CPMV 100 µg per mouse) or once (1 mg CPMV and 1 mg CPMV in the described CPMV‐G4 assembly). CPMV‐G4 was vortexed immediately prior to injection. Prior to treatment group assignment, total luminescence was determined on day seven and used to match total cancer burden between treatment groups (*n* = 5–6).

For biodistribution studies, mice were injected with 2 million ID8‐*Defb29/Vegf‐Luc* cells intraperitoneally and cancer cells were allowed to grow for 1 week. Following establishment of intraperitoneal disease as determined by IVIS imaging, mice were injected with PBS, 1 mg of AF647‐CPMV, or 1 mg of AF647‐CPMV‐G4. Mice were imaged prior to injection, immediately after injection, and at times 1 h, 3h, 6 h, 12 h, 24 h, 48 h, 72 h, 7 d, 11 , and 14 d. Images were obtained in Spectral unmixing mode and each time‐point was unmixed to isolate AF647 signal using the same library; an unmixing library was established separately for each cage immediately following injection. ROI analysis was performed on unmixed images from each time‐point and radiant efficiency ((p s^−1^ cm^−2^)/(µW cm^−2^)). Graphed relative fluorescence intensities are a summary of two successive biodistribution studies of *n* = 1 followed by *n* = 2.

## Conflict of Interest

The authors declare no conflict of interest.

## Supporting information

Supplementary
