# Supplementary material for: Slow‐Release Formulation of Cowpea Mosaic Virus for In Situ Vaccine Delivery to Treat Ovarian Cancer
Source: Adv Sci (Weinh). 2018 Feb 21;5(5):1700991. doi: 10.1002/advs.201700991 (PMC5979803; doi:10.1002/advs.201700991)
Supplement: Supplementary file 1 — Supplementary [file ADVS-5-1700991-s001.pdf]

Copyright WILEY-VCH Verlag GmbH & Co. KGaA, 69469 Weinheim, Germany, 2018.

ADVANCED SCIENCE
Open Access

# Supporting Information

for *Adv. Sci.,* DOI: 10.1002/advs.201700991

# Slow-Release Formulation of Cowpea Mosaic Virus for In Situ Vaccine Delivery to Treat Ovarian Cancer

*Anna E. Czapar, Brylee David B. Tiu, Frank A. Veliz, Jonathan K. Pokorski, and Nicole F. Steinmetz**

Copyright WILEY-VCH Verlag GmbH & Co. KGaA, 69469 Weinheim, Germany, 2016.

# Supporting Information

**Slow-release formulation of cowpea mosaic virus for *in situ* vaccine delivery to treat ovarian cancer**

*Anna E Czapar[1#], Brylee David B Tiu[2#$], Frank A Veliz[2], Jonathan K. Pokorski[5], Nicole F Steinmetz[2,3,4,5,6]**

#Both authors contributed equally to this work.

E-mail: * nicole.steinmetz@case.edu

Departments of [1]Pathology, [2]Biomedical Engineering, [3]Materials Science and Engineering, [4]Radiology, [5]Macromolecular Science and Engineering, [6]Case Comprehensive Cancer Center, Division of General Medical Sciences-Oncology, Case Western Reserve University, 2109 Adelbert Road, Cleveland, Ohio 44106, USA

[$]present address: Department of Bioengineering, University of California, Berkeley, California, USA 94720.

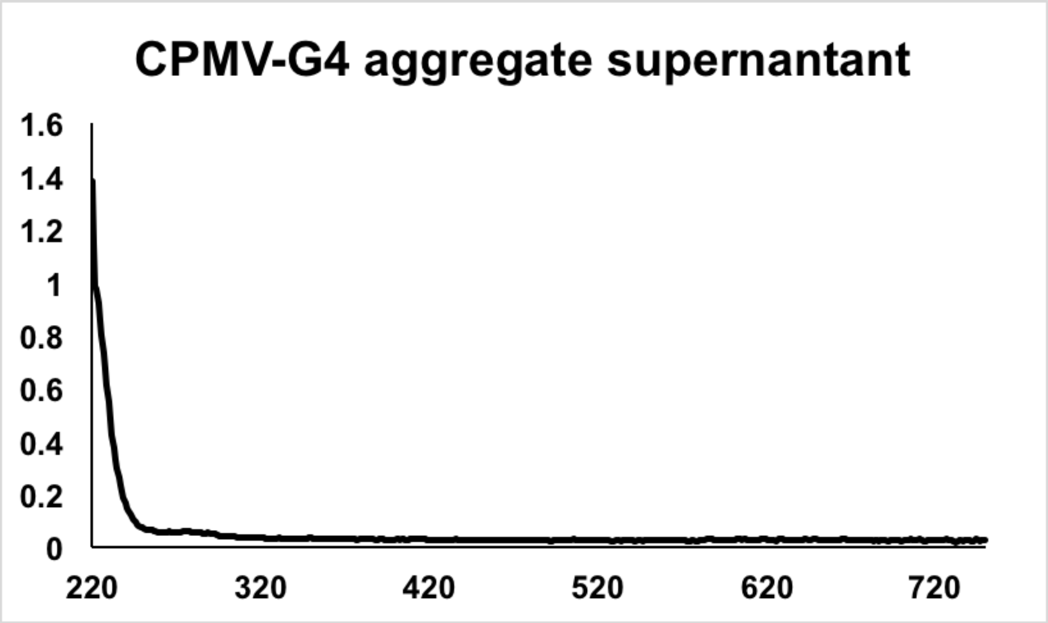


**Figure S1.** UV-vis spectrum of supernatant following brief centrifugation of CPMV-G4 at 2.5 mg/mL concentration in 25 mM NaCl (see also Figure 1E).

**Table S1.** Dynamic light scattering data for CPMV-G4 assemblies in different NaCl concentrations.

| [NaCl] (mM) | $R_{H,1}$ (nm) | PDI of $R_{H,1}$ | %Mass of $R_{H,1}$ | $R_{H,2}$ (nm) | PDI of $R_{H,2}$ | %Mass of $R_{H,2}$ | $R_{H,3}$ (nm) | PDI of $R_{H,3}$ | %Mass of $R_{H,3}$ |
|---|---|---|---|---|---|---|---|---|---|
| 0 | 71.00 ± 5.15 | 0.1 ± 0.02 | 55.59 ± 10.68 | 840.61 ± 362.74 | 0.16 ± 0.14 | 44.41 ± 10.68 | - | - | - |
| 10 | 14.14 ± 1.59 | 0.01 ± 0 | 0.54 ± 0.19 | 205.05 ± 86.34 | 0.01 ± 0.01 | 1.78 ± 1.22 | 1605.58 ± 85.73 | 0.1 ± 0.05 | 97.95 ± 1.47 |
| 25 | 220.24 ± 133.55 | 0.01 ± 0 | 2.52 ± 3.01 | 1272.52 ± 111.7 | 0.13 ± 0.1 | 97.48 ± 3.01 | - | - | - |
| 50 | 209.25 ± 25.73 | 0.02 ± 0.01 | 1.35 ± 0.44 | 1714.86 ± 193.92 | 0.35 ± 0.12 | 98.65 ± 0.44 | - | - | - |
| 100 | 26.99 ± 1.79 | 0.01 ± 0.01 | 0.34 ± 0.14 | 216.67 ± 149.01 | 0.04 ± 0.03 | 3.85 ± 3.83 | 1553.37 ± 471.64 | 1.08 ± 1.12 | 96.94 ± 3.81 |
| 150 | 16.35 ± 0.65 | 0.02 ± 0.01 | 4.01 ± 0.85 | 905.65 ± 309.99 | 0.31 ± 0.25 | 95.98 ± 0.85 | - | - | - |
| 200 | 11.77 ± 3.59 | 0.02 ± 0.01 | 21.61 ± 12.59 | 352.82 ± 17.21 | 0.47 ± 0.12 | 78.39 ± 12.59 | - | - | - |
| 300 | 13.42 ± 1.28 | 0.05 ± 0.05 | 24.07 ± 1.09 | 210.82 ± 16.73 | 0.31 ± 0.15 | 75.93 ± 1.09 | - | - | - |
| 350 | 10.03 ± 3.24 | 0.01 ± 0.01 | 24.60 ± 4.14 | 25.04 ± 0.53 | 0.01 ± 0.01 | 5.54 ± 0.21 | 184.4 ± 2.56 | 0.07 ± 0.07 | 71.7 ± 6.29 |
| 400 | 11.55 ± 0.30 | 0.01 ± 0 | 25.37 ± 1.27 | 192.13 ± 5.31 | 0.26 ± 0.03 | 74.63 ±1.27 | - | - | - |

$R_H$ – Average hydrodynamic radius for the smallest distribution. $R_{H,1}$, $R_{H,2}$, and $R_{H,3}$ are average values of the hydrodynamic radius distributions present in the sample. If only 2 distributions are present, $R_{H,3}$ is not defined.

**PDI of $R_H$** – Polydispersity index of an $R_H$ distribution.

**%Mass of $R_H$** – Mass percent of an $R_H$ distribution based on the total mass of aggregates detected.

**Table S2.** Dynamic light scattering data for CPMV-G4 assemblies in different PBS concentrations.
These PBS concentrations were selected to match the concentration of NaCl in the DLS experiment in NaCl environment (see Table S1).

| [PBS] (×) | [NaCl] (mM) | $R_{H,1}$ (nm) | PDI of $R_{H,1}$ | %Mass of $R_{H,1}$ | $R_{H,2}$ (nm) | PDI of $R_{H,2}$ | %Mass of $R_{H,2}$ | $R_{H,3}$ (nm) | PDI of $R_{H,3}$ | %Mass of $R_{H,3}$ |
|---|---|---|---|---|---|---|---|---|---|---|
| 0 | 0 | 71.00 ± 5.15 | 0.1 ± 0.02 | 55.59 ± 10.68 | 840.61 ± 362.74 | 0.16 ± 0.14 | 44.41 ± 10.68 | - | - | - |
| 0.07 | 9.59 | 402.93 ± 187.44 | 0.02 ± 0.02 | 4.74 ± 3.72 | 2032.75 ± 614.81 | 0.11 ± 0.14 | 95.24 ± 3.71 | 48.55 ± 17.09 | 0.005 ± 0.008 | 0.03 ± 0.02 |
| 0.18 | 24.66 | 116.69 ± 77.29 | 0.02 ± 0.02 | 0.35 ± 0.47 | 1343.95 ± 238.05 | 0.33 ± 0.27 | 99.65 ± 0.47 | - | - | - |
| 0.36 | 49.32 | 76.94 ± 86.16 | 0.03 ± 0.04 | 0.37 ± 0.17 | 1458.63 ± 503.72 | 1.02 ± 0.86 | 99.75 ± 0.25 | - | - | - |
| 0.73 | 100 | 24.25 ± 2.14 | 0.01 ± 0.01 | 1.28 ± 0.49 | 729.74 ± 136.65 | 0.21 ± 0.08 | 98.72 ± 0.49 | - | - | - |
| 1.09 | 149.33 | 17.53 ± 7.07 | 0.05 ± 0.03 | 9.39 ± 8.22 | 378.49 ± 87.90 | 0.40 ± 0.25 | 83.63 ± 12.05 | - | - | - |
| 1.82 | 249.34 | 13.14 ± 4.05 | 0.005 ± 0.006 | 21.66 ± 7.54 | 296.81 ± 2.26 | 0.07 ± 0 | 77.57 ± 8.62 | - | - | - |
| 2.19 | 300 | 11.98 ± 0.68 | 0.01 ± 0.01 | 24.76 ± 1.31 | 55.90 ± 28.54 | 0.02 ± 0 | 1.26 ± 0.46 | 304.33 ± 12.56 | 0.05 ± 0.03 | 73.98 ± 1.77 |
| 2.55 | 349.35 | 9.81 ± 4.47 | 0.01 ± 0.01 | 29 ± 5.76 | 274.62 ± 16.37 | 0.07 ± 0.04 | 67.28 ± 8.95 | 25.34 ± 7.7 | 0.02 ± 0.03 | 5.58 ± 3.46 |
| 2.92 | 400 | 4.90 ± 2.99 | 0.01 ± 0 | 37.28 ± 5.23 | 18.96 ± 6.13 | 0.05 ± 0 | 9.40 ± 5.83 | 280.13 ± 7.34 | 0.18 ± 0.07 | 53.32 ± 11.06 |
| 3.28 | 449.36 | 3.88 ± 2.09 | 0.01 ± 0.01 | 54.12 ± 15.93 | 23.69 ± 2.46 | 0.01 ± 0.01 | 3.47 ± 2.68 | 250.34 ± 0.49 | 0.01 ± 0 | 39.18 ± 17.82 |
| 3.65 | 500 | 2.46 ± 0.81 | 0 | 65.26 ± 1.19 | 14.24 ± 7.30 | 0.01 ± 0 | 7.1 ± 3.85 | 247.10 ± 2.65 | 0.02 ± 0 | 27.21 ± 5.64 |

$\boldsymbol{R_H}$ – Average hydrodynamic radius for the smallest distribution. $\boldsymbol{R_{H,1}}$, $\boldsymbol{R_{H,2}}$, and $\boldsymbol{R_{H,3}}$ are average values of the hydrodynamic radius distributions present in the sample. If only 2 distributions are present, $\boldsymbol{R_{H,3}}$ is not defined.
**PDI of $\boldsymbol{R_H}$** – Polydispersity index of an $R_H$ distribution.
**%Mass of $\boldsymbol{R_H}$** – Mass percent of an $R_H$ distribution based on the total mass of aggregates detected.
